# Analysis of correlation between initial alveolar bone density and apical root resorption after 12 months of orthodontic treatment without extraction

**DOI:** 10.1590/2176-9451.19.5.097-102.oar

**Published:** 2014

**Authors:** Paula Cabrini Scheibel, Adilson Luiz Ramos, Lilian Cristina Vessoni Iwaki, Kelly Regina Micheletti

**Affiliations:** 1 MSc in Integrated Dentistry, State University of Maringá, UEM; 2 Adjunct professor, Department of Dentistry, State University of Maringá, UEM; 3 Associate professor of Dental radiology and Stomatology, Department of Dentistry, State University of Maringá, UEM; 4 PhD resident in Orthodontics, State University of São Paulo, UNESP

**Keywords:** Root resorption, Tooth movement, Dental radiography, Bone density

## Abstract

**OBJECTIVE:**

The aim of the present study was to investigate the correlation between initial alveolar bone density of upper central incisors (ABD-UI) and external apical root resorption (EARR) after 12 months of orthodontic movement in cases without extraction.

**METHODS:**

A total of 47 orthodontic patients 11 years old or older were submitted to periapical radiography of upper incisors prior to treatment (T_1_) and after 12 months of treatment (T_2_). ABD-UI and EARR were measured by means of densitometry.

**RESULTS:**

No statistically significant correlation was found between initial ABD-UI and EARR at T_2_ (r = 0.149; p = 0.157).

**CONCLUSION:**

Based on the present findings, alveolar density assessed through periapical radiography is not predictive of root resorption after 12 months of orthodontic treatment in cases without extraction.

## Abstract (pt)

**OBJETIVO:**

avaliar a correlação entre a densidade óssea alveolar inicial dos incisivos centrais superiores (DOA-IS) e a reabsorção radicular apical externa (RRAE) após 12 meses de movimentação ortodôntica em casos sem extração.

**MÉTODOS:**

quarenta e sete pacientes ortodônticos (maiores que 11 anos) foram submetidos ao exame periapical dos incisivos superiores no pré-tratamento (T_1_) e 12 meses após (T_2_). Mensurou-se a RRAE no intervalo de 12 meses, bem como a densidade óssea alveolar inicial da região apical desses dentes por meio da fotodensitometria.

**RESULTADOS:**

não houve correlação estatisticamente significativa entre a DOA-IS inicial e a RRAE em T_2_ (r = 0,149; p = 0,157).

**CONCLUSÃO:**

a densidade alveolar avaliada pela radiografia periapical não se apresentou como fator de interferência ou preditivo para reabsorção radicular após 12 meses de tratamento ortodôntico sem extração.

## INTRODUCTION

Orthodontic movement often results in external apical root resorption (EARR).^1^^-^ 6 While this event does not significantly affect teeth support in most patients, severe root resorption occurs in 5% to 14.5% of cases.^1^^-^ 5

As the risk factors identified for EARR stemming from orthodontic treatment have limited effectiveness, studies involving multivariate analyses have suggested that individual factors may contribute to the etiology of this condition.^1^^,^ 3 ^,^ 4 ^,^ 7 ^,^ 8 ^,^ 9 This belief has led researchers to investigate the influence of maxillary bone density. Kaley and Phillips^10^ as well as Horiuchi et al^11^ found that dental movement in areas of greater bone density, such as cortical bone, is associated with greater root resorption. Goldie and King^12^ found that low bone mineral density (BMD) in rats induced by lactation and calcium deficiency (increased secretion of parathyroid hormone) led to less root resorption during orthodontic movement in comparison to the control group. However, Otis et al^13^ found no significant effect of alveolar bone density around roots over the amount of root resorption.

Considering the divergent results of previous studies, the aim of the present investigation was to test the hypothesis that increased alveolar bone density is an individual predisposing factor for EARR during orthodontic treatment, especially in cases without extraction.

## MATERIAL AND METHODS

A prospective study was carried out with a sample of 91 upper incisors in 47 patients, 11 years old or older who had participated in a previous study.^6^ All patients had a complete fixed appliance installed with straight-wire orthodontics at the clinics of the Orthodontic Postgraduate Program of the State University of Maringá and at Maringá Dental Association (Brazil) between July 2008 and April 2009. In selecting the sample, the following inclusion criteria were applied: Signed informed consent form; patients who were 11 years old or older; fully intact crown of upper incisors or only with proximal restorations; and scheduled orthodontic treatment without extractions and ⁄or incisor intrusion. The exclusion criteria were: Previous history of fixed orthodontic treatment; previous root resorption; history of dentoalveolar trauma to upper incisors; history of osteoporosis or rickets and hyperparathyroidism.

All procedures were approved by the State University of Maringá Institutional Review Board, Brazil (190 ⁄ 2008). Each volunteer was submitted to periapical radiography of the upper incisor region either immediately prior to or immediately after bracket bonding (T_1_), as well as 12 months after orthodontic treatment (T_2_). Radiographs were taken using the RX Timex 70 C (Gnatus, Ribeirão Preto, SP, Brazil) and Pro 70-Intra (Prodental, Ribeirão Preto, São Paulo, Brazil) x-ray equipment operating with 70 kVp, 7 mA and a 0.25-second exposure time.^6^ A five-step 2 x 20 x 3.5 mm aluminum wedge (Al step-wedge) was attached to the apical region perpendicular to the film (Agfa Dentus M2 "Comfort"). Kodak developing and fixing solutions (Kodak do Brazil, Comércio e Indústria Ltda, São José dos Campos, SP, Brazil) were used to develop the radiographs. The radiographic film was processed manually using the time-temperature method.^14^ Development time was determined after verifying the liquid temperature (2 minutes in developer with temperature between 20 and 26^o^C). Intermediate washing was standardized at 30 seconds and fixing time was standardized at 10 minutes.^15^ Radiographic images were digitized using a scanner under 400 ppi resolution (ArtixScan 18000F, Microtek, São Paulo, SP, Brazil).

### Measure of alveolar bone density 

**Figure 1:**
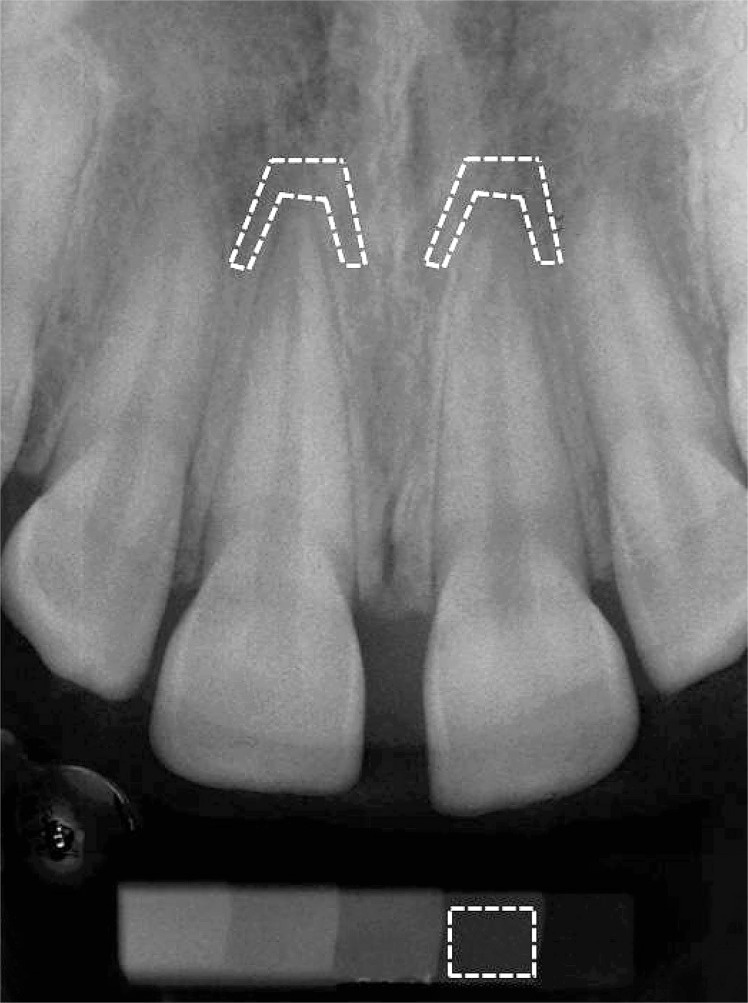
Scanned periapical radiograph; region of interest selected in the apical region of upper central incisors and second step of aluminum stepwedge (evaluated by histogram tool of Adobe Photoshop CS2 program).

By means of the histogram tool provided by Photoshop CS3 software (Adobe System, California, USA), a trapezoidal region of interest was outlined in the alveolar bone process of the apical region of upper central incisors to estimate optical density expressed in grey level values ranging from 0 (black) to 255 (white).^14^ Each trapezoidal region of interest consisted of approximately 2000 pixels and was selected in such a way so as to avoid roots, lamina dura and nasal spine. The digital reading of each step was performed by selecting a rectangular trapezoidal region of interest of approximately 2500 pixels (Fig 1). Using the optical densities of aluminum step wedge, mean optical density of bone between both central incisors was converted into millimeters aluminum equivalent (mmAl / Eq).

### Measure of external apical root resorption

**Figure 2:**
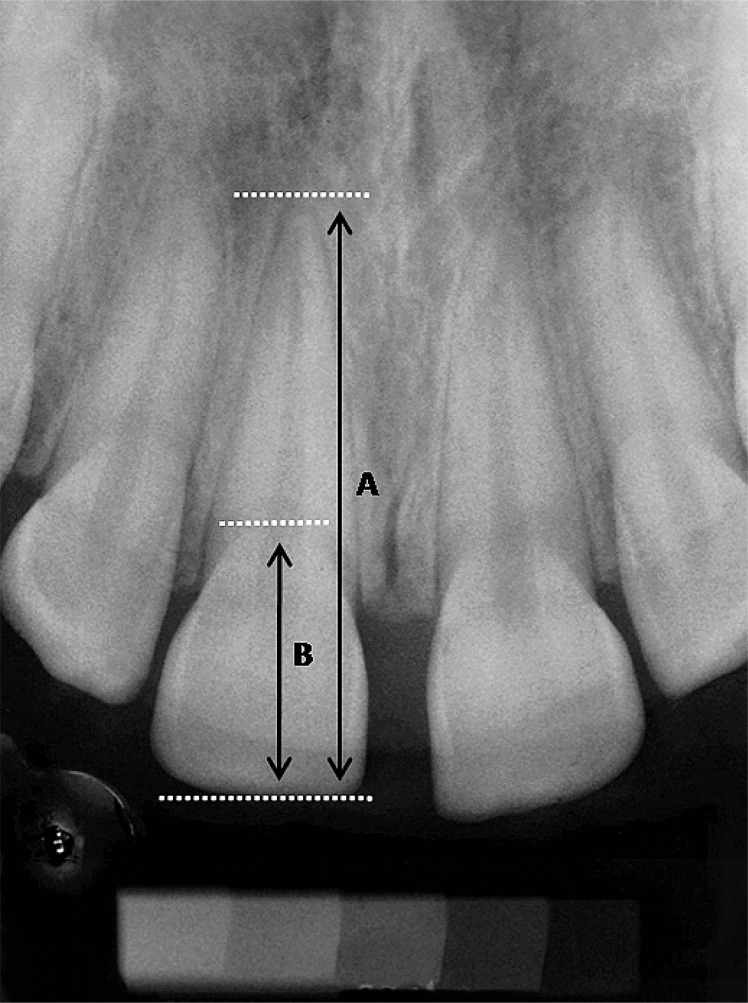
Radiograph illustrating measures: (A) incisal-apical distance (tooth length) used to calculate root resorption; (B) distance from incisal edge to cementum-enamel junction (crown length) used for correction of radiograph inclination.

Tooth length (TL) and crown length (CL) of upper incisors (#11 and #21) at both evaluation times (TLT_1_ and TLT_2_; CLT_1_ and CLT_2_) were measured at a precision of 0.1 mm with the aid of CorelDRAW X4 software.^6^^,^ 16 ^,^ 17 ^,^ 18 These measures corresponded to the distance from the incisal edge to the root apex and the greatest distance between the incisal edge and the cementum-enamel junction. The long axis of the tooth was used as reference (Fig 2). To compensate for possible variations in inclination during radiograph taking at different times (presuming that the crown measure remains unaltered during treatment),^17^^,^ 19 ^,^ 20 expected tooth length at T_2_ was calculated using the following equation:^6^^,^ 18 ^,^ 21

**TLT_2_ expected **= CLT_2_ . TLT_1_/CLT_1_

The amount of EARR was determined by subtracting expected tooth length at T_2_ by tooth length at T_2_: 

**EARR T_2_** = TLT**_2_** expected - TLT_2_

The amount of EARR was expressed in percentage in relation to initial tooth length. 0% resorption was classified as absent; 1 to 4% was classified as rounding of roots; 4 to 8% was classified as mild; and 8 to 12% was classified as moderate.^6^ Intra-examiner reliability was statistically assessed by analyzing the differences between duplicate measures on the radiographic images of 25 randomly selected patients (tooth and crown length, optical densities of the alveolar bone and second step of the aluminum wedge) with a 15-day interval between measures at both T_1 _and T_2_. The error of the method was calculated using Dahlberg's formula:



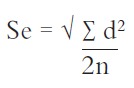



In which 'd' is the difference between pairs of measurements and 'n' is the number of pairs of measurements. Spearman's correlation coefficient (r) was also employed. Although no statistically significant differences were found between the first and second measures, the mean of each variable was used in the subsequent statistical tests to minimize random error.

### Statistical analysis

Neither EARR nor ABD-UI had normal distribution (Lilliefors test). Thus, nonparametric Spearman correlation test was used to determine potential correlations between initial ABD-UI and EARR at T_2_. Significance level was set at 5% (P < 0.05) for all statistical tests.

## RESULTS

No significant differences were found between teeth #11 and #21 regarding EARR and ABD-UI. After 12 months of treatment, mean EARR was 3.5% (standard deviation: 3.03%; range: 0 to 12.1%) (Table 1). Three patients (6%) had no root resorption; 18 patients (38%) had resorption between 1 and 4%; 18 patients (38%) had resorption between 4 and 8%; and eight patients (17%) had resorption between 8 and 12% (Table 2). No statistically significant correlation was found between initial ABD-UI and EARR after 12 months (r = 0.149; p = 0.157).

**Table 1:** Descriptive characterization of sample (n = 47) according to age, mean percentage of EARR after 12 months of treatment and initial ABD-UI in mmEq/Al of 91 upper incisors.

	Minimum	Maximum	Mean ± SD
Age (years)	11	51	20 ± 10.52
EARR (%)	0	12.1	3.5 ± 3.03
ABD-UI (mmEq/Al)	1.24	4.97	2.55 ± 0.89

**Table 2:** Descriptive characterization of sample (n = 47) according to percentage of EARR in more resorbed upper central incisors.

EARR (%)	Patients n (%)
0%	3 (6%)
≥ 1 and ≤ 4%	18 (38%)
> 4 and ≤ 8%	18 (38%)
> 8 and ≤ 12%	8 (17%)
	47 (100%)

## DISCUSSION

Apical root resorption can occur in the early stages of orthodontic treatment, especially in upper incisors which generally undergo greater movement in comparison to other teeth.^3^^,^ 8 ^,^ 9 ^,^ 17 ^,^ 20 The degree of compression of periodontal ligament is believed to influence the extent of EARR, as greater compression is accompanied by an increase in the area of hyalinization and, theoretically, an increase in EARR severity. However, the force produced by an orthodontic appliance is not necessarily the same force distributed along the periodontal ligament. A number of aspects influence the degree of final root compression and consequent tissue damage, such as mechanical factors (direction of movement; duration and intensity of force applied) and biological factors (crown to root ratio, root anatomy and density of trabecular bone).^21^

Periapical radiography is the method of choice to assess apical root resorption stemming from orthodontic treatment, mainly due to the cost-benefit ratio of this method. Periapical radiographs are known to have greater reliability in comparison to lateral and panoramic radiographs.^22^ However, periapical radiographs have less sensitivity and specificity in comparison to volumetric tomography. The three-dimensional visualization of teeth is the major advantage of computed tomography over conventional radiographic exams,^23^^,^ 24 but the disadvantages of this method are greater cost and greater exposure to x-rays in comparison to periapical radiography.

In previous studies employing dual-energy x-ray absorptiometry (DXA), systemic BMD (lumbar spine and femur) was not correlated with maxillomandibular alveolar BMD.^14^^,^ 25 However, Scheibel and Ramos^25^ found a correlation between alveolar bone density in the region of upper incisors and neck of the femur using periapical radiography. Other studies have also found a correlation between systemic BMD and alveolar bone mass assessed by periapical radiography and expressed in mmEqAl.^26^^-^ 29 Thus, the choice of periapical radiography is based on the possibility of selecting trabecular alveolar bone and avoiding the lamina dura, roots and other structures in comparison to DXA.

A study investigating alveolar density of anterior and posterior regions of the maxilla and mandible found that only the densities of the anterior maxilla and posterior mandible were correlated.^25^ This study and other investigations therefore suggest the specific densitometric evaluation of the region of interest.^30^^,^ 31 Anterior alveolar regions of the maxilla and mandible have greater densitometric values in comparison to posterior regions,^14^^,^ 31 ^,^ 32 which may be related to the greater occurrence of root resorption in upper incisors together with other factors such as root anatomy and orthodontic mechanics.

The dentoalveolar complex of each patient is unique in terms of size, orientation and density, and associations between EARR and alveolar density and morphology have not yet been established.^13^ In the literature, only Otis et al^13^ performed a direct investigation on these associations. Using digital techniques on cephalometric radiographs, the authors measured the dimensions of lower incisors and surrounding bone structures (quantitative aspect) as well as density of trabecular bone (qualitative aspect). The present study also investigated the correlation between alveolar bone mass and EARR; however, the methodologies differed with regard to the region examined, type of radiographic exam and methods employed to determine bone density. In the present study, no significant correlation was found between ABD-UI and EARR 12 months after orthodontic treatment. Similarly, setting aside methodological differences, Otis et al^13^ found that the amount of alveolar bone adjacent to the root, cortical bone thickness, trabecular bone density and fractal dimension were not significantly correlated with the extent of EARR.

As cortical bone is denser than trabecular bone, a number of studies have investigated associations between bone density and root resorption in an indirect manner by analyzing the proximity of roots and cortical bone during orthodontic movement.^1^^,^ 10 ^,^ 11 ^,^ 33 In a histological study involving monkeys, Wainwright^33^ found no differences in the amount of root resorption between movement against cortical bone and trabecular bone. A clinical study also found no greater root resorption in patients with roots and apices subjectively judged to be in close proximity with palatal cortical bone.^1^

Kaley and Philips^10^ studied a case series of 200 patients submitted to orthodontic treatment with the edgewise technique. The authors reported findings that contrast those of the studies cited above. Six patients (3%) had severe resorption (greater than one quarter of the length of the root) in both upper central incisors. For other teeth, this extent of resorption occurred in less than 1% of patients. Using a case-control model, the characteristics of 21 patients with severe resorption were compared to randomly selected controls from the same case series. Risk factors of root resorption related to orthodontic treatment included increased proximity of maxillary incisors roots to palatal cortical bone (odds ratio: 20), maxillary surgery (odds ratio: 8) and root torque (odds ratio: 4.5). According to the authors, proximity of roots to palatal cortical bone may be directly related to other statistically significant measures observed in the study, such as torque of upper incisors, changes in angle, duration of use of rectangular arch wires and extractions in the upper arch.

Horiuchi et al^11^ suggest that proximity of upper central incisors roots to palatal cortical bone during orthodontic treatment may explain approximately 12% of variation in root resorption, whereas alveolar bone thickness explains about 2%. The authors also state that tooth extrusion and lingualization of the crown also contribute to root resorption. In another study, the amount of incisor movement was significantly correlated to the amount of EARR, with even greater movement in cases in which prior extraction of premolars was performed (r = 0.61; P < 0.05).^34^

It makes sense to measure the total displacement of a tooth by the root apex which is where pathological resorption occurs.^35^ In a number of studies, apical displacement, especially in the anteroposterior direction and against cortical bone, was found to be significantly correlated with apical root resorption.^1^^,^ 10 ^,^ 11 In a meta-analysis,^35^ mean apical resorption was correlated with apical displacement (r = 0.822) and total treatment duration (r = 0.852). However, prolonged treatment alone did not appear to be related to greater root resorption. Although certain procedures, such as torque of upper incisors, changes in angle, duration of rectangular arch wires and extractions in the upper arch, are not found to be direct factors, they seem to be correlated with greater EARR.^10^ The findings suggest the need for further investigations of this possible association with larger samples and cases involving greater movement, such as cases with extraction and Class II malocclusion.

## CONCLUSION

Based on the present findings, alveolar density in the apical region of upper incisors assessed by means of periapical radiographs is not predictive of root resorption 12 months after orthodontic treatment in cases without extraction.
